# Sex‐Specific Differences in Dietary Iron Intake and Sleep Disorder in NHANES 2005–2014 Population

**DOI:** 10.1002/fsn3.71627

**Published:** 2026-03-08

**Authors:** Xinping Yu, Baowen Fan, Heqing Zheng, Mingxu Liu, Lanxiang Wu, Sheng Tian, Wei Wu

**Affiliations:** ^1^ Department of Neurology, the Second Affiliated Hospital, Jiangxi Medical College Nanchang University Nanchang China; ^2^ Department of Neurosurgery, the Second Affiliated Hospital, Jiangxi Medical College Nanchang University Nanchang Jiangxi China

**Keywords:** cross‐sectional study, iron intake, L‐shaped, sleep disorder, women

## Abstract

The association between dietary iron intake and sleep disorder remains insufficiently understood. The aim of this research was to explore the relationship between dietary iron intake and sleep disorder. This research used data from the National Health and Nutrition Examination Survey conducted during the period from 2005 to 2014. Weighted logistic regression analyses were performed to detect the association between iron intake and sleep disorder. The participants had a mean age of 46.92 ± 0.29 years, and 51.19% were female. Overall, dietary iron intake showed a significant inverse association with sleep disorders (log_2_‐transformed iron intake: OR, 0.84; 95% CI: 0.74, 0.96). Per log_2_‐transformed unit increase in iron intake, the odds of sleep disorders decreased by 23% in women (OR: 0.77; 95% CI: 0.66, 0.91). However, dietary iron intake was not associated with sleep disorder in men (OR: 0.94; 95% CI: 0.81, 1.10). In addition, there was an L‐shaped relationship between iron intake with sleep disorder among women (*p* for non‐linearity = 0.01). This study demonstrated that higher dietary iron intake was inversely associated with sleep disorder exclusively in women.

## Graphical abstract

This study reveals sex‐specific differences in the relationship between dietary iron intake and sleep disorder. Increased iron intake is associated with a lower prevalence of sleep disorders, with this correlation observed exclusively in women. The analysis also shows an L‐shaped association in women, indicating a threshold effect.

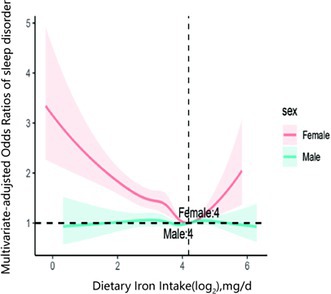

## 1 Introduction

Sleep is a physiologically reversible state of physical and psychiatric rest, characterized by essential active processes that occupy roughly one‐third of human life (Carden 2020). Sleep disorders are prevalent conditions that disrupt normal circadian rhythms, negatively affecting mental and physical health. They can manifest as insufficient sleep, excessive sleep, or abnormal movements during sleep. Sleep disorders are linked not only to diminished quality of living and work performance, but also to increased physical and mental health issues (Medic et al. 2017). It is recognized as a risk factor for numerous ailments, including cardiovascular events, metabolic disease, immune dysfunction, neurological disorders, and even elevated mortality rates (Fishbein et al. 2021; Grandner and Fernandez 2021; Smiley et al. 2019).

Numerous researchers have explored the potential reasons and risk factors behind sleep disorders, such as inadequate sleep practices, racial inequalities and various lifestyle elements, with a particular focus on dietary habits. Epidemiological observations have established a correlation between sleep disorders and nutrient intake. Micronutrient deficiencies are common in healthy individuals. A growing body of research has identified connections between dietary macronutrient intake and sleep quality (Ikonte et al. 2019). For example, high dietary glycemic index diets were associated with a higher incidence of insomnia, whereas greater intakes of dietary fiber, whole grains, and non‐juice fruits predicted fewer insomnia symptoms and more restorative sleep (Gangwisch et al. 2020). Iron deficiency stands as the most predominant micronutrient deficiency globally, affecting approximately two billion people (German and Juul 2021). Iron, an integral part of the diet of all living organisms, is indispensable for numerous critical functions including oxygen transport, cellular respiration, immunity, neurotransmitter activity, and DNA synthesis (Malik et al. 2025). Dietary iron is absorbed by duodenal epithelial cells and tightly regulated by iron‐storage proteins and receptors to balance intake and utilization. Iron deficiency has been proven to be associated with sleep‐disordered breathing, attention‐deficit/hyperactivity disordered sleep problems, restless sleep disorder and general sleep disturbance (Leung et al. 2020). Given the importance of iron for sleep quality, it is essential to determine how dietary iron relates to sleep disorders.

To our knowledge, no previous study has examined the association between dietary iron intake and sleep disorders. Therefore, the present study was designed to investigate this relationship. Additionally, it aimed to assess gender differences in how dietary iron influences sleep disorders.

## 2 Materials and Methods

### 2.1 Study Population

This study is a cross‐sectional analysis of data from the 2005 to 2014 cycles of the National Health and Nutrition Examination Survey (NHANES). NHANES is an ongoing nationwide health survey conducted by the National Center for Health Statistics. Data were collected in two phases: an in‐person interview and a physical examination conducted at the Mobile Examination Centre (MEC).

### 2.2 Definition of Sleep Disorder

Following published methods (Chunnan et al. 2022), sleep disorder status was determined by self‐report responses in the NHANES questionnaire. Participants who answered “yes” to the question “Have you ever been told by a doctor or other health professional that you have a sleep disorder?” were classified as having a sleep disorder.

### 2.3 Dietary Iron Assessment

Dietary iron intake data were obtained from a 24‐h food recall survey. Intake values were calculated with the US Department of Agriculture's Food and Nutrient Database for Dietary Studies. Detailed dietary interview procedures are available in the NHANES Dietary Interview Procedures Manual.

### 2.4 Covariate Assessment

Covariates were assessed through the in‐person interview and included age, sex, race, educational attainment, marital status, and poverty income ratio (PIR). Educational attainment was categorized as less than high school, high school, or beyond high school. Marital status was classified into married/living with a partner, separated/divorced/widowed, or never married. The PIR was grouped into three classes: ≤ 1.3, 1.3–3.5 and > 3.5. Both smoking and drinking status were divided into never (reference), former, or current. Body mass index (BMI) was calculated as dividing weight (in kilograms) by the square of height (in meters) and is classified into three categories: less than 25.0, 25.0 to less than 30.0, and 30.0 or greater kg/m^2^. Hypertension was diagnosed by self‐reported use of currently prescribed antihypertensive medications or by physician diagnosis. History of stroke was defined on the basis of self‐reported physician‐diagnosed conditions.

### 2.5 Statistical Analysis

Following the NHANES analytic guidelines, all analyses incorporated sample weights, stratification, and clustering to take into account complicated sampling designs. Continuous variables were displayed as mean ± standard error, while categorical variables were displayed as percentages and frequencies. One‐way ANOVA for continuous variables and chi‐square test for categorical variables were applied to compare differences between groups.

In view of the skewed distribution of iron intake, a log2 transformation was applied for the regression analysis (Figure S1). Participants were then divided into quartiles based on their iron intake. Multivariate logistic regression was used to estimate the link between iron intake and sleep disorder, with results presented as odds ratios (ORs) and 95% confidence intervals (CIs). The interaction between dietary iron intake and gender on the risk of sleep disorder was assessed with a stratified multivariate logistic regression model. Restricted cubic spline was applied to evaluate the dose–response relationship. The threshold value was identified with a two‐piecewise logistic regression model.

All data analysis and processing were performed with R statistical software (version 4.4.1). All tests were two‐tailed, and *p*‐values < 0.05 were considered statistically significant.

## 3 Results

### 3.1 Baseline Characteristic

**FIGURE 1:**
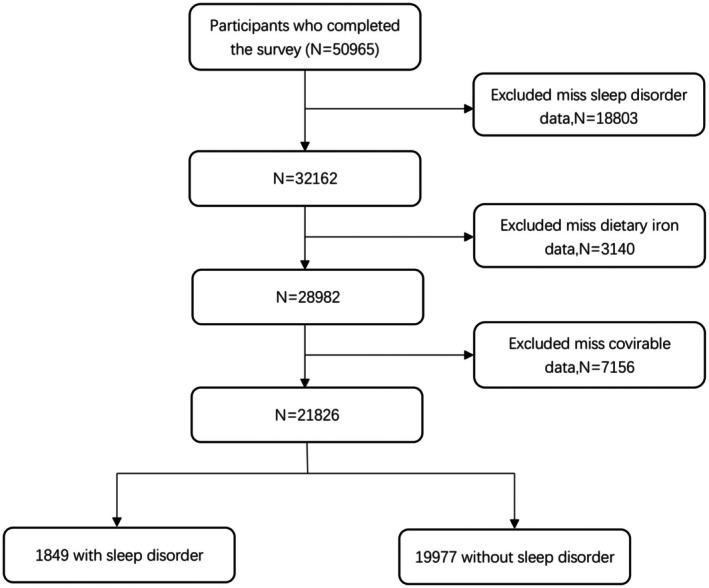
Flow chart of participants inclusion and exclusion for analysis.

Figure 1 illustrates the process of participant recruitment and the inclusion/exclusion criteria in the study. A total of 21,826 participants were included in the final analysis. Baseline characteristics of study participants by dietary iron intake quartiles are presented in Table 1. Overall, the mean age was 46.92 ± 0.29 years, and 51.19% of participants were male. The ranges of dietary iron intake for quartiles 1–4 were ≤ 9.14, 9.15–13.07, 13.08–18.67 and > 18.67 mg/d, respectively. Participants in the highest quartile were more likely to be younger, male, non‐Hispanic White, married/living with partner, never smoking, current drinking, no hypertension, no stroke and had a high PIR and educational level.

**TABLE 1:** Baseline characteristics by categories of the study participants.

Characteristic	Dietary iron intake quartiles (mg/d)					*p*
	Total	Q1	Q2	Q3	Q4	
Age, years	46.92 ± 0.29	47.85 ± 0.34	48.04 ± 0.36	46.85 ± 0.36	45.18 ± 0.44	< 0.001
Sex, *n* (%)						
Male	10,757 (48.81)	1842 (31.31)	2400 (41.54)	2922 (52.94)	3593 (66.35)	< 0.001
Female	11,069 (51.19)	3624 (68.69)	3050 (58.46)	2531 (47.06)	1864 (33.65)	
Race, *n* (%)						
Mexican American	3295 (7.77)	736 (6.84)	817 (7.58)	866 (8.10)	876 (8.41)	< 0.001
Non‐Hispanic Black	4586 (10.70)	1405 (14.10)	1159 (11.04)	1043 (9.37)	979 (8.79)	
Non‐Hispanic White	10,411 (70.75)	2389 (67.33)	2549 (70.35)	2650 (71.61)	2823 (73.21)	
Other Hispanic	1801 (4.67)	512 (5.27)	465 (4.67)	422 (4.56)	402 (4.28)	
Other Race	1733 (6.10)	424 (6.46)	460 (6.35)	472 (6.36)	377 (5.32)	
Marital status, *n* (%)						
Married/Living with partner	13,108 (63.93)	2945 (60.10)	3284 (65.11)	3418 (67.26)	3461 (68.30)	< 0.001
Separated/Divorced/Widowed	4115 (16.13)	1278 (21.14)	1081 (17.93)	926 (14.75)	830 (12.96)	
Never married	3905 (17.71)	1024 (18.76)	908 (16.96)	956 (17.99)	1017 (18.74)	
Education level, *n* (%)						
Below High School	2094 (5.04)	652 (6.32)	516 (4.93)	475 (4.65)	451 (4.42)	< 0.001
High School	8307 (34.21)	2261 (39.17)	2100 (34.84)	1969 (31.25)	1977 (32.26)	
Above High School	11,425 (60.75)	2553 (54.50)	2834 (60.23)	3009 (64.10)	3029 (63.32)	
PIR, *n* (%)						
Low	7085 (21.90)	2084 (27.50)	1762 (21.71)	1567 (18.47)	1672 (20.59)	< 0.001
Medium	6384 (27.25)	1642 (29.89)	1618 (27.78)	1600 (26.25)	1524 (25.47)	
High	8357 (50.86)	1740 (42.62)	2070 (50.50)	2286 (55.28)	2261 (53.94)	
BMI, kg/m^2^						
< 25.0	6385 (30.60)	1580 (31.48)	1562 (29.70)	1599 (30.18)	1644 (31.08)	< 0.001
25.0 to < 30.0	7294 (33.48)	1709 (30.84)	1847 (34.40)	1828 (32.82)	1910 (35.54)	
≥ 30.0	8147 (35.92)	2177 (37.68)	2041 (35.89)	2026 (37.00)	1903 (33.39)	
Smoking status, *n* (%)						
Never	11,748 (54.12)	2971 (52.28)	2895 (53.32)	3001 (56.12)	2881 (54.49)	< 0.001
Former	5431 (24.81)	1168 (21.71)	1401 (25.66)	1423 (25.53)	1439 (25.99)	
Current	4647 (21.07)	1327 (26.01)	1154 (21.02)	1029 (18.35)	1137 (19.52)	
Drinking status, *n* (%)						
Never	2951 (10.69)	886 (12.79)	746 (11.01)	705 (10.33)	614 (8.97)	< 0.001
Former	4123 (15.58)	1131 (17.21)	1032 (15.67)	982 (14.68)	978 (14.97)	
Current	14,752 (73.73)	3449 (70.01)	3672 (73.31)	3766 (74.99)	3865 (76.06)	
Stroke, *n* (%)						
No	20,985 (97.09)	5171 (95.68)	5226 (96.60)	5276 (97.82)	5312 (98.02)	< 0.001
Yes	841 (2.91)	295 (4.32)	224 (3.40)	177 (2.18)	145 (1.98)	
Hypertension, *n* (%)						
No	14,111 (68.51)	3334 (65.26)	3505 (68.57)	3536 (68.19)	3736 (71.54)	< 0.001
Yes	7715 (31.49)	2132 (34.74)	1945 (31.43)	1917 (31.81)	1721 (28.46)	
Sleep disorder, *n* (%)						
No	19,977 (91.32)	4974 (89.94)	4987 (91.19)	5025 (92.31)	4991 (91.67)	0.01
Yes	1849 (8.68)	492 (10.06)	463 (8.81)	428 (7.69)	466 (8.33)	
Energy (kcal/d)	2196.19 ± 10.62	1358.48 ± 10.98	1907.38 ± 10.88	2360.31 ± 13.78	3015.00 ± 21.25	< 0.001
Protein (g/d)	84.00 ± 0.44	50.45 ± 0.41	72.13 ± 0.50	90.79 ± 0.58	116.86 ± 0.95	< 0.001
Carbohydrate intake (g/d)	262.41 ± 1.30	164.12 ± 1.33	223.92 ± 1.55	277.21 ± 1.69	366.97 ± 2.68	< 0.001
Fat consumption (g/d)	83.60 ± 0.54	49.33 ± 0.56	73.88 ± 0.60	91.58 ± 0.72	113.98 ± 1.08	< 0.001

*Note:* Continuous variables were shown as mean ± standard error, and *p* value was calculated by weighted one‐way analyses of variance. Categories variables were shown as percentage, and *p* value was calculated by weighted *χ* ^2^ test.

The finding from the univariate analysis showed that factors such as age, sex, ethnicity, BMI, PIR, marital status, smoking status, alcohol intake, and history of hypertension and stroke were correlated with sleep disorder (Table S1).

### 3.2 Association Between Dietary Iron Intake and Sleep Disorder

**FIGURE 2:**
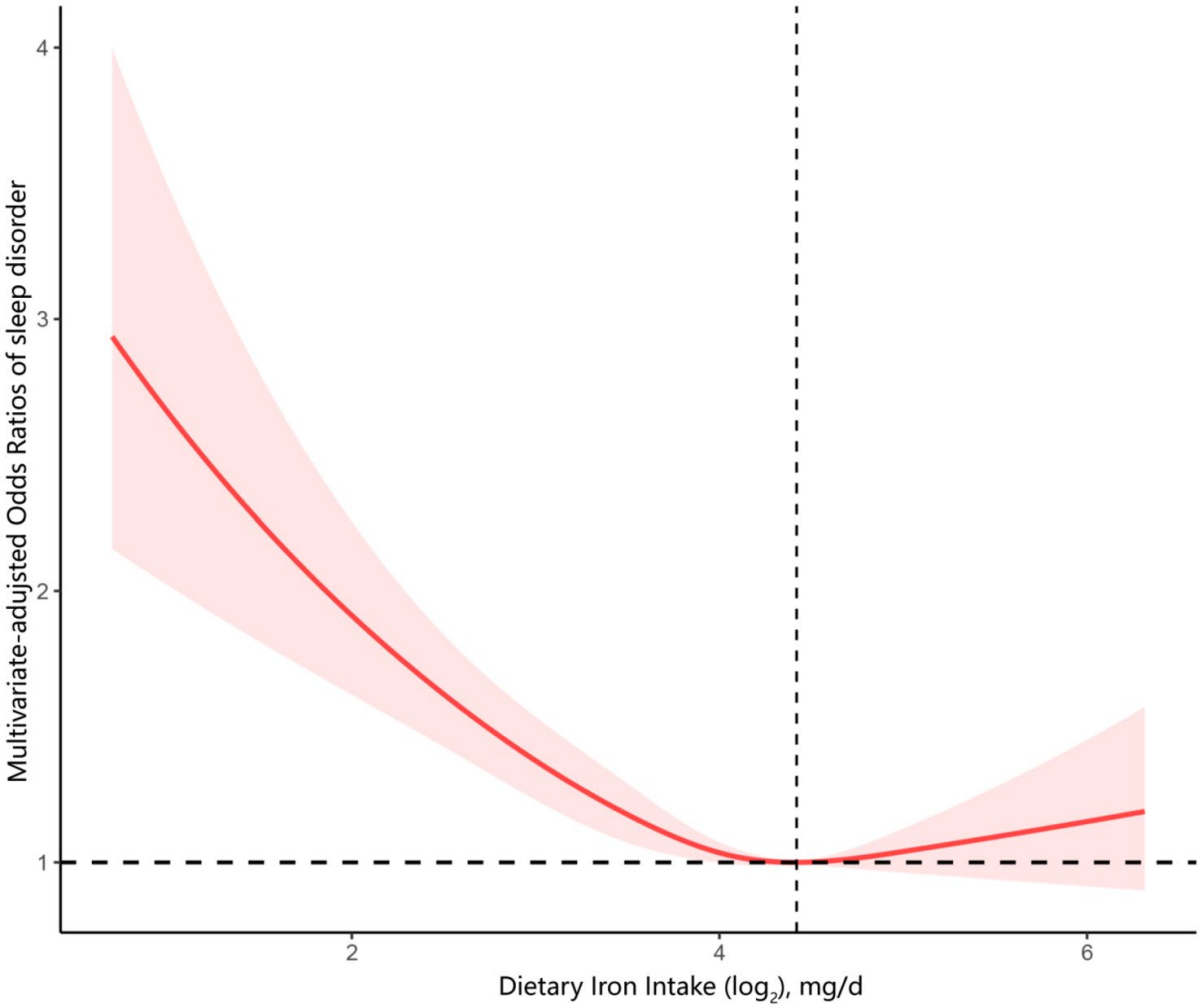
Association between dietary iron intake and sleep disorder odds ratio. The model was adjusted for age, sex, race, marital status, educational attainment, PIR, BMI, smoking status, drinking status, stroke, hypertension, energy consumption, protein consumption, carbohydrate consumption, and fat consumption.

The association between dietary iron intake and sleep disorder was estimated using multiple logistic regression analyses. The increase in iron intake was inversely related to the prevalence of sleep disorders across all three models (Table 2). Compared with the Q1 reference group, the odds of sleep disorder in the fully adjusted model were 0.81 (95% CI: 0.67, 0.97) for Q2, 0.68 (95% CI: 0.54, 0.85) for Q3, and 0.73 (95% CI: 0.57, 0.94) for Q4. To investigate the dose–response correlation between dietary iron intake and sleep disorder, we conducted restricted cubic spline analysis, as illustrated in Figure 2. In the fully adjusted RCS curve, the relationship between dietary iron intake and sleep disorder was non‐linear (*p* for non‐linearity = 0.01).

**TABLE 2:** Association between dietary iron intake and sleep disorder in different models.

	Model 1		Model 2		Model 3	
	OR (95% CI)	*p*	OR (95% CI)	*p*	OR (95% CI)	*p*
Continuous	0.90 (0.82, 0.98)	0.02	0.88 (0.80, 0.96)	0.01	0.84 (0.74, 0.96)	0.01
Categories						
Q1	1.00 (Reference)		1.00 (Reference)		1.00 (Reference)	
Q2	0.86 (0.72, 1.03)	0.10	0.84 (0.70, 1.00)	0.05	0.81 (0.67, 0.97)	0.02
Q3	0.75 (0.62, 0.90)	0.003	0.72 (0.59, 0.87)	0.001	0.68 (0.54, 0.85)	< 0.001
Q4	0.81 (0.67, 0.99)	0.04	0.78 (0.63, 0.95)	0.02	0.73 (0.57, 0.94)	0.02
*p* for trend		0.02		0.01		0.01

*Note:* Model 1 was adjusted for nothing. Model 2 was additionally adjusted for age and sex. Model 3 was additionally adjusted for model 2+ race, marital status, education attainment, BMI, PIR, drinking status, smoking status, stroke, hypertension, energy consumption, protein consumption, carbohydrate consumption, and fat consumption.

Abbreviations: BMI, body mass index; CI, confidence interval; OR, odds ratio; PIR, poverty income ratio.

As shown in Table 3, gender differences were evident: the association between dietary iron intake and sleep disorder was observed only in women. Across all three models, the risk of sleep disorders was lower in women than in men with increasing dietary iron intake (*p* for interaction = 0.02). Similarly, when iron intake was examined by sex using categorical variables, the results remained consistent. Fully adjusted regression analyses for women and men separately are presented in Figure S2. Compared with the Q1 group, the odds of sleep disorder among women were significantly lower in Q2 (OR: 0.79; 95% CI: 0.61, 1.03), Q3 (OR: 0.60; 95% CI: 0.43, 0.84), and Q4 (OR: 0.66; 95% CI: 0.44, 0.97) (*p* for trend = 0.01). In contrast, no association between iron intake and sleep disorders was found in men, regardless of whether iron was modeled as a categorical or continuous variable. Figure S3 illustrates the dose–response relationship between iron intake and sleep disorder in men and women respectively. Among female participants, the relationship was non‐linear (*p* for non‐linearity = 0.01), whereas no such pattern was observed in male participants.

**TABLE 3:** Association between dietary iron intake and sleep disorder in different models among male and female.

	Model 1		Model 2		Model 3	
	OR (95% CI)	*p*	OR (95% CI)	*p*	OR (95% CI)	*p*
Female						
Continuous	0.79 (0.71, 0.88)	< 0.001	0.79 (0.71, 0.88)	< 0.001	0.77 (0.66, 0.91)	0.003
Categories						
Q1	1.00 (Reference)		1.00 (Reference)		1.00 (Reference)	
Q2	0.80 (0.65, 0.98)	0.03	0.80 (0.65, 0.98)	0.03	0.79 (0.61, 1.03)	0.08
Q3	0.60 (0.47, 0.78)	< 0.001	0.60 (0.47, 0.78)	< 0.001	0.60 (0.43, 0.84)	0.004
Q4	0.67 (0.51, 0.87)	0.004	0.68 (0.52, 0.89)	0.01	0.66 (0.44, 0.97)	0.04
*p* for trend		< 0.001		< 0.001		0.01
Male						
Continuous	0.95 (0.84, 1.07)	0.39	0.98 (0.86, 1.11)	0.73	0.94 (0.81, 1.10)	0.46
Categories						
Q1	1.00 (Reference)		1.00 (Reference)		1.00 (Reference)	
Q2	0.94 (0.70, 1.27)	0.69	0.94 (0.69, 1.27)	0.66	0.88 (0.64, 1.21)	0.43
Q3	0.85 (0.65, 1.12)	0.25	0.88 (0.67, 1.15)	0.34	0.79 (0.59, 1.07)	0.13
Q4	0.86 (0.64, 1.16)	0.31	0.91 (0.68, 1.23)	0.54	0.83 (0.61, 1.14)	0.24
*p* for trend		0.24		0.52		0.20
*p* for interaction		0.01		0.01		0.02

*Note:* Model 1 was adjusted for nothing. Model 2 was additionally adjusted for age. Model 3 was additionally adjusted for model 2+ race, marital status, education attainment, BMI, PIR, drinking status, smoking status, stroke, hypertension, energy consumption, protein consumption, carbohydrate consumption and fat consumption.

Abbreviations: BMI, body mass index; CI, confidence interval; OR, odds ratio; PIR, poverty income ratio.

**FIGURE 3:**
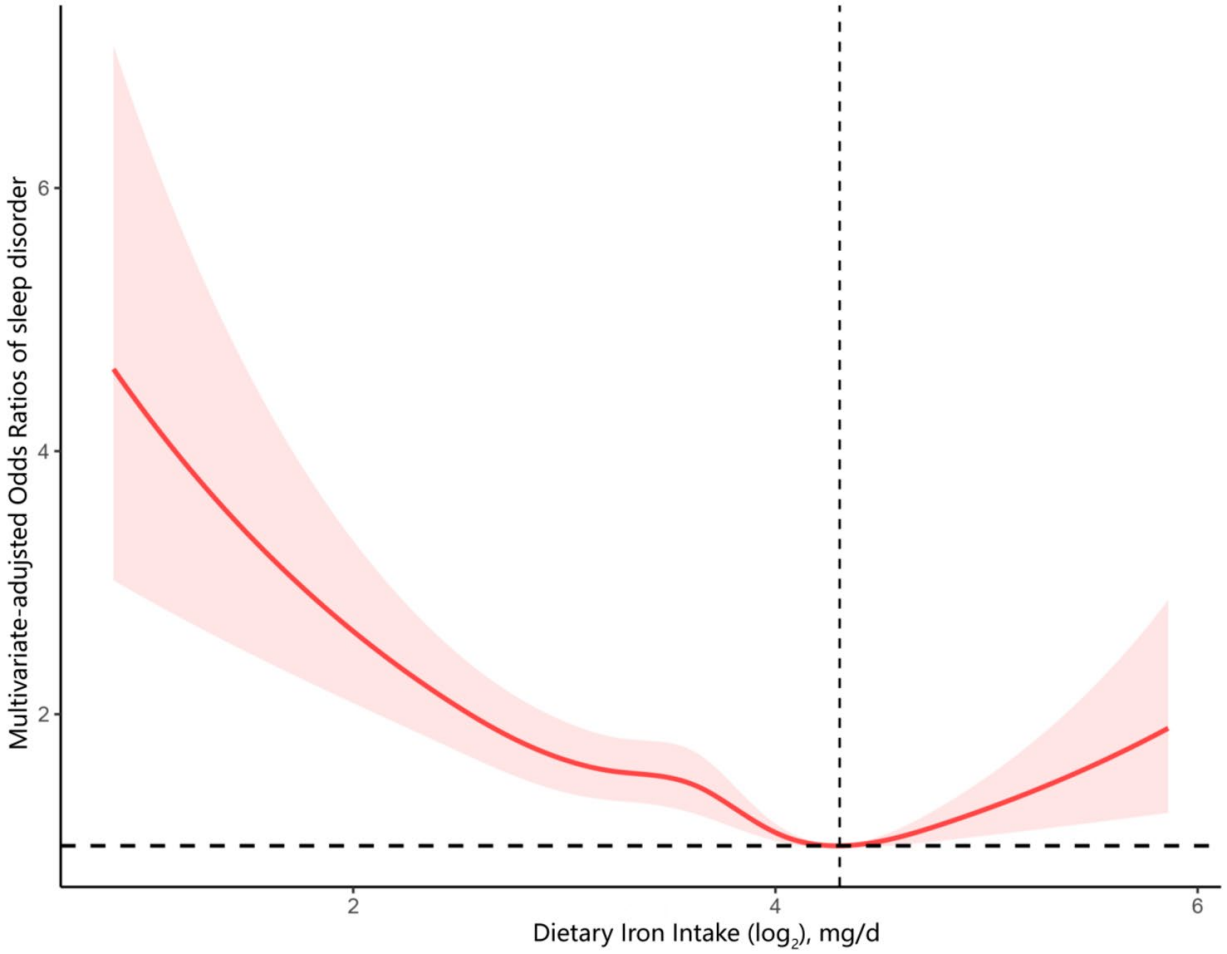
Association between dietary iron intake and sleep disorder odds ratio among females. The model was adjusted for age, race, marital status, education attainment, PIR, BMI, smoking status, drinking status, stroke, hypertension, energy consumption, protein consumption, carbohydrate consumption, and fat consumption.

Based on the above findings, we further examined the association between dietary iron intake and sleep disorders in female participants (Figure 3). The relationship was non‐linear and displayed an L‐shaped curve, with the inflection point at 4.19 mg/d for log_2_‐transformed iron intake. Table 4 summarizes the results from the two‐piecewise logistic regression model fitted with a recursive algorithm. To the right of the inflection point, the OR was 1.03 (95% CI: 0.70, 1.50) with a *p*‐value of 0.89, indicating no significant association. To the left, however, iron intake showed a significant negative correlation with sleep disorder. The OR was 0.81 (95% CI: 0.68, 0.97) with a *p*‐value of 0.02. Thus, an intake of approximately 4.19 mg/day (log_2_‐transformed) corresponded to the lowest prevalence of sleep disorders in women.

**TABLE 4:** Threshold effect analysis of iron on sleep disorder using two‐piecewise linear regression model among female.

	Model 1		Model 2		Model 3	
	OR (95% CI)	*p*	OR (95% CI)	*p*	OR (95% CI)	*p*
< Inflection point	0.84 (0.74, 0.96)	0.01	0.84 (0.74, 0.96)	0.01	0.81 (0.68, 0.97)	0.02
≥ Inflection point	1.22 (0.83, 1.51)	0.44	1.16 (0.86, 1.57)	0.34	1.03 (0.70, 1.50)	0.89

*Note:* Model 1 was adjusted for nothing. Model 2 was additionally adjusted for age. Model 3 was additionally adjusted for model 2+ race, marital status, education attainment, BMI, PIR, drinking status, smoking status, stroke, hypertension, energy consumption, protein consumption, carbohydrate consumption, and fat consumption.

Abbreviations: BMI, body mass index; CI, confidence interval; OR, odds ratio; PIR, poverty income ratio.

### 3.3 Sensitivity Analysis

After excluding participants with extremely high energy intake, 21,354 individuals remained. The association between iron intake and sleep disorder remained significant. Compared with the lowest quartile (Q1), the odds ratios for sleep disorders in Q2, Q3, and Q4 were 0.84 (95% CI: 0.70–1.02; *p* = 0.07), 0.72 (95% CI: 0.57–0.91; *p* = 0.01), and 0.77 (95% CI: 0.60–0.99; *p* = 0.05) (Table S2). Gender‐specific differences in this association were also evident (*p* for interaction = 0.01; Table S3). Among women, ORs relative to Q1 were 0.83 (95% CI: 0.64–1.08) for Q2, 0.64 (95% CI: 0.46–0.91) for Q3, and 0.68 (95% CI: 0.46–1.01) for Q4, with a significant dose–response trend (*p* for trend = 0.03). In men, dietary iron intake was not related to sleep disorder, whether analyzed as categorical or continuous variable in the fully adjusted model (Table S3).

## 4 Discussion

To our knowledge, this is the first large‐scale, nationally representative study to report a significant inverse association between dietary iron intake and sleep disorder prevalence, which was observed only in women. Restricted cubic spline analysis further revealed an L‐shaped, non‐linear dose–response curve among female participants, consistent with a threshold effect beyond which protection plateaued. In contrast, no parallel pattern was detected in men. Thus, the findings underscore that the link between dietary iron intake and sleep disorders is inherently gender‐specific.

To date, several studies have examined the connection between iron intake and sleep‐related problems. A cross‐sectional analysis of an online survey among health, non‐pregnant Saudi adults aged 25–55 years revealed that lower iron status was associated with poorer sleep quality (Al‐Khudhairy et al. 2021). Another study utilized actigraphy to measure sleep quality in adolescents and young women, finding that individuals with lower sleep efficiency had markedly lower iron intake than those with higher efficiency (Hashimoto et al. 2020). A longitudinal study of elite female athletes found that each 1‐mg increase in iron intake increased sleep duration by 0.55 min (Condo et al. 2022). A retrospective study of Angelman syndrome patients aged < 18 years indicated that sleep difficulties are partly related to iron deficiency, and iron supplementation can modestly alleviate these problems (Ryan et al. 2020). In addition, a prospective single‐center randomized controlled trial in Austria showed that iron supplementation evidently improves the severity of sleep quality (Macher et al. 2020). Collectively, these findings suggest that iron deficiency can contribute to sleep issues and that iron repletion may enhance sleep quality, underscoring the pivotal role of iron in sleep health.

This study identified a significant inverse association between dietary iron intake and sleep disorder that persisted after adjustment for potential confounders. The mechanism underlying this inverse relationship remains unclear, yet our results accord with current biological evidence. Iron is crucial for the normal functioning of numerous enzymes and proteins and its deficiency can induce diffuse and subtle alterations in the central nervous system through multiple pathways. First, iron exerts complex effects on dopaminergic signaling as a cofactor of the dopamine D2 receptor (Daubian‐Nosé et al. 2023). Iron deficiency can impair dopamine neurotransmission in certain areas of the brain, including regions crucial to sleep regulation (Quiroz et al. 2016). Dopaminergic neuromodulation plays a critical role in sleep control (Feld and Born 2020). Iron serves as a cofactor for the enzymes that synthesize and degrade γ‐aminobutyric acid (GABA) (Ferreira et al. 2019). GABA is the brain's principal inhibitory neurotransmitter and is essential for both the initiation and maintenance of sleep (Lee et al. 2025). Iron deficiency can disturb GABA metabolism, raising cortical excitability and making it harder to relax and fall asleep. Serotonin also participates in the regulation of the sleep–wake cycle, and its synthesis likewise depends on iron‐containing enzymes (Ferreira et al. 2019; Lee et al. 2025). By disrupting these monoaminergic systems, iron deficiency may further fragment sleep architecture—for example, reducing deep sleep or increasing nocturnal awakenings. Furthermore, iron is implicated in myelination process and the metabolic activity of neurons. Because of the need for sufficient iron for proper myelination and dendritic formation, changes induced by iron‐ deficiency may affect the functionality of various brain systems, including those responsible for generating sleep spindles observable (Peirano et al. 2007). Spindles have been proved to be generated by synchronous activities of the functional neural network connecting the thalamus and the cortex (Szalárdy et al. 2024). Iron is necessary to ensure the regular functioning of the oscillating thalamocortical network.

Building upon the inverse association, we further investigated potential sex differences and observed distinct variations between males and females. Among women, dietary iron intake had an inverse association with sleep disorder, meaning that higher iron intake was linked to a lower risk of sleep disorder. However, this pattern was not observed in men. This sex‐specific disparity plausibly reflects both physiological and hormonal factors. Menstrual blood loss and the high iron demands of pregnancy substantially increase the risk of iron depletion in women, thereby heightening their vulnerability to the neurological sequelae of inadequate iron stores (Sholzberg et al. 2025). Consequently, women's heightened vulnerability to iron deficiency may amplify the impact of dietary iron on sleep disorders. Men, who typically maintain larger systemic iron reserves and rarely experience deficiency, showed no such relationship in the present analysis (Kiss 2015). Additionally, underlying hormonal divergence may drive sex‐specific effect. Studies have shown that estrogen concentrations are positively correlated with systemic iron release and with increased iron requirements (Badenhorst et al. 2022). This correlation implies that estrogen might influence systemic iron metabolism. Conversely, men generally exhibit larger iron reserves than women that may be attributable to higher circulating testosterone concentrations. Testosterone suppresses heparin in a dose‐dependent manner in males, thereby enhancing intestinal iron absorption and increasing systemic iron availability (Badenhorst et al. 2022). Thereby, sex‐specific hormonal profiles drive distinct iron‐metabolism set‐points, which in turn may determine the degree to which dietary iron intake confers protection against sleep disorder in women but not in men.

Our analysis further revealed a significant non‐linear relationship between dietary iron intake and the prevalence of sleep disorder among women, manifesting as a characteristic L‐shaped curve. This pattern indicates a sharp decrease in the risk of sleep disorders as iron intake increases from deficient levels, followed by a plateau where additional intake confers no further protective benefit. This non‐linear relationship suggests that there is an optimal range of iron intake within which the prevalence of sleep disorder is minimized. This finding underscores the importance of maintaining an appropriate balance in iron intake to optimize sleep health, particularly in women. Biologically, this non‐linearity may be explained by the dual role of iron in neurophysiological processes critical to sleep regulation. Iron serves as an essential cofactor for enzymes involved in the synthesis of key neurotransmitters such as dopamine and serotonin, both of which regulate sleep–wake cycles (Ipsiroglu et al. 2024). Inadequate iron availability likely impairs monoamine synthesis and synaptic signaling, increasing susceptibility to sleep disturbances which predominates at low intake levels (Murat et al. 2015). By contrast, upon reaching a threshold, iron‐dependent enzymatic activity likely saturates, and neuronal requirements are met. Beyond this point, excess iron may not provide additional benefit and could even promote oxidative stress via Fenton reactions, potentially explaining the flat trajectory of the curve (Huang et al. 2025).

Our findings carry significant clinical and public health implications, particularly for women. These findings suggest that maintaining adequate dietary iron intake may serve as a valuable non‐pharmacological strategy for improving sleep health among women. The observed non‐linear relationship between iron intake and sleep disorder underscores the necessity of achieving a balanced iron status. It is crucial to maintain optimal iron levels to minimize the risk of sleep disorders. Clinicians are encouraged to perform dietary assessments and to evaluate iron status in women who present with sleep complaints. Indiscriminate iron supplementation in the absence of confirmed deficiency is not recommended, as it may induce oxidative stress and systemic inflammation that could further disrupt sleep. Rather, a balanced diet that sustains optimal iron levels should be promoted. Such an approach supports overall health, prevents the neurological consequences associated with both low and high iron status, and ultimately contributes to improving sleep quality in women.

There are several merits to our research that should be considered when evaluating the findings. Firstly, this research was based on a sizable and demographically diverse sample of various ethnicities and socioeconomic statuses, thereby mirroring the broader United States adult population. Secondly, the investigation identified sex‐specific differences in dietary iron intake and sleep disorders, detecting the association exclusively among women and not among men. This distinction enables clinicians to implement more personalized prevention strategies. Furthermore, the study revealed a non‐linear association between dietary iron intake and sleep disorders, highlighting the importance of maintaining an optimal iron intake for sleep health. This finding underscores the necessity of balanced nutrition to prevent sleep disturbances.

There are several limitations to this study. First, the cross‐sectional design precludes definitive inference regarding the directionality of the association between dietary iron intake and sleep disorders. Hence, reverse causation or bidirectional effects cannot be ignored. Second, random measurement error is likely unavoidable because only a single 24‐h dietary recall was used, thereby failing to capture intra‐individual variation in nutrient intake. Moreover, the study was conducted in an American population, and the generalisability of the findings to other ethnic or geographic groups remains to be established. Future investigations should therefore replicate these observations in diverse populations to validate the results and to clarify their global relevance.

## 5 Conclusions

The cross‐sectional study indicated an inverse, non‐linear association between dietary iron intake and the prevalence of sleep disorder. Furthermore, a statistically significant interaction by sex was observed: the non‐linear relationship between dietary iron intake and sleep disorders was evident exclusively among women and was absent among men.

## Author Contributions

**Xinping Yu:** methodology (lead), software (lead), writing – original draft (lead), writing – review and editing (equal). **Baowen Fan:** writing – review and editing (lead). **Heqing Zheng:** writing – review and editing (equal). **Mingxu Liu:** formal analysis (supporting), writing – review and editing (equal). **Lanxiang Wu:** data curation (supporting), resources (equal). **Sheng Tian:** data curation (supporting), resources (equal). **Wei Wu:** funding acquisition (lead), project administration (lead), writing – review and editing (lead).

## Funding

This work was supported by the National Natural Science Foundation of China (Grant numbers: 82160227 and 82360234), Natural Science Foundation of Jiangxi Province (Grant numbers: 20252BAC200479), National Nature Incubation Project of the Second Affiliated Hospital of Nanchang University (Grant numbers: 2023YNFY12019 and 2024YNFY12028), and Internal funding project of the Second Affiliated Hospital of Nanchang University (Grant numbers: 2022efyC02).

## Ethics Statement

Study protocols for NHANES were granted approval by the NCHS ethics review board (Protocol #2005–06 Protocol, Protocol #2011–17 and Protocol #2018–01).

## Consent

The authors have nothing to report.

## Conflicts of Interest

The authors declare no conflicts of interest.

## Supporting information


**Appendix S1:** fsn371627‐sup‐0001‐AppendixS1.docx.

## Data Availability

All data generated or analyzed during this study is included at this URL https://wwwn.cdc.gov/nchs/nhanes/Default.aspx.
