# Supplementary material for: Sex‐Specific Differences in Dietary Iron Intake and Sleep Disorder in NHANES 2005–2014 Population
Source: Food Sci Nutr. 2026 Mar 8;14(3):e71627. doi: 10.1002/fsn3.71627 (PMC12967456; doi:10.1002/fsn3.71627)
Supplement: Supplementary file 1 — Appendix S1: fsn371627‐sup‐0001‐AppendixS1.docx. [file FSN3-14-e71627-s001.docx]

**Supplementary Material**

**Sex-specific differences in the effect of the dietary iron intake and sleep disorder in the NHANES 2005-2014 population**

Xinping Yu^1.2*^ , Heqing Zheng^1.2^ , Mingxu Liu^1.2^ , Sheng Tian^1.2#^ , Lanxiang Wu^1.2#^ ,Wei Wu^1.2#^

**Supplementary Table 1.** Relationship of covariates and sleep disorder.

**Supplementary Table 2.** Association between dietary iron intake and sleep disorder in participants with extreme energy intake was not included.

**Supplementary Table 3.** Association between dietary iron intake and sleep disorder in participants among male and female with extreme energy intake was not included.

**Supplementary Figure 1.** Distribution of dietary iron intake in the study population

**Supplementary Figure 2.** Relative odds of sleep disorder according to dietary iron quartile among male and female.

**Supplementary Figure 3.** Association between dietary iron intake and sleep disorder odds ratio among female and male.

| **Supplementary Table 1.** Relationship of covariates and sleep disorder. | | | |
| --- | --- | --- | --- |
| Variable | OR | 95% CI | *P* value |
| Age,years | 1.02 | 1.01,1.02 | <0.0001 |
| Sex |  |  |  |
| Male | 1.00(Reference) |  |  |
| Female | 0.83 | 0.74,0.93 | 0.002 |
| Race |  |  |  |
| Mexican American | 1.00(Reference) |  |  |
| Non-Hispanic Black | 1.6 | 1.28,2.00 | <0.0001 |
| Non-Hispanic White | 1.89 | 1.54,2.33 | <0.0001 |
| Other Hispanic | 1.67 | 1.26,2.21 | <0.001 |
| Other Race | 1.33 | 1.02,1.74 | 0.04 |
| Marital status |  |  |  |
| Married/Living with partner | 1.00(Reference) |  |  |
| Separated/Divorced/Widowed | 1.23 | 1.05,1.44 | 0.01 |
| Never married | 0.66 | 0.54,0.80 | <0.0001 |
| Education level |  |  |  |
| Below High School | 1.00(Reference) |  |  |
| High School | 1.22 | 0.98,1.53 | 0.08 |
| Above High School | 1.2 | 0.98,1.47 | 0.08 |
| PIR |  |  |  |
| Low | 1.00(Reference) |  |  |
| Medium | 0.85 | 0.70,1.02 | 0.07 |
| High | 0.81 | 0.70,0.94 | 0.01 |
| BMI, kg/m^2^ |  |  |  |
| < 25.0 | 1.00(Reference) |  |  |
| 25.0 to < 30.0 | 1.44 | 1.18,1.77 | <0.001 |
| ≥ 30.0 | 3.62 | 3.08,4.27 | <0.0001 |
| Smoking status |  |  |  |
| Never | 1.00(Reference) |  |  |
| Former | 1.66 | 1.42,1.94 | <0.0001 |
| Current | 1.49 | 1.30,1.71 | <0.0001 |
| Drinking status |  |  |  |
| Never | 1.00(Reference) |  |  |
| Former | 1.78 | 1.41,2.25 | <0.0001 |
| Current | 1.05 | 0.85,1.30 | 0.66 |
| Stroke |  |  |  |
| No | 1.00(Reference) |  |  |
| Yes | 2.67 | 2.13,3.35 | <0.0001 |
| Hypertension |  |  |  |
| No | 1.00(Reference) |  |  |
| Yes | 2.57 | 2.30,2.88 | <0.0001 |

Abbreviations: PIR, poverty income ratio; BMI, body mass index; OR, odds ratio; CI, confidence interval.

| **Supplementary Table 2.** Association between dietary iron intake and sleep disorder in participants with extreme energy intake was not included. | | | | | | |
| --- | --- | --- | --- | --- | --- | --- |
|  | Model 1 | | Model 2 | | Model 3 | |
|  | OR(95%CI) | *P* value | OR(95%CI) | *P* value | OR(95%CI) | *P* value |
| Continuous | 0.88(0.81,0.97) | 0.01 | 0.86(0.79,0.95) | 0.003 | 0.86(0.75,0.98) | 0.03 |
| Categories |  |  |  |  |  |  |
| Q1 | 1.00(Reference) |  | 1.00(Reference) |  | 1.00(Reference) |  |
| Q2 | 0.88(0.73,1.05) | 0.15 | 0.85(0.71,1.02) | 0.09 | 0.84(0.70,1.02) | 0.07 |
| Q3 | 0.76(0.63,0.93) | 0.01 | 0.73(0.60,0.89) | 0.003 | 0.72(0.57,0.91) | 0.01 |
| Q4 | 0.80(0.66,0.97) | 0.03 | 0.76(0.62,0.93) | 0.01 | 0.77(0.60,0.99) | 0.05 |
| P for trend |  | 0.01 |  | 0.003 |  | 0.03 |

Abbreviations: PIR, poverty income ratio; BMI, body mass index; OR, odds ratio; CI, confidence interval.

Model 1 was adjusted for nothing.

Model 2 was additionally adjusted for age and sex.

Model 3 was additionally adjusted for model 2+ race, marital status, education attainment, BMI, PIR, drinking status, smoking status, stroke, hypertension,energy consumption, protein consumption, carbohydrate consumption and fat consumption.

| **Supplementary Table 3.** Association between dietary iron intake and sleep disorder in participants among male and female with extreme energy intake was not included. | | | | | | | | |
| --- | --- | --- | --- | --- | --- | --- | --- | --- |
|  | Model 1 | | Model 2 | | | Model 3 | | |
|  | OR(95%CI) | *P* value | | OR(95%CI) | *P* value | | OR(95%CI) | *P* value |
| Female |  |  | |  |  | |  |  |
| Continuous | 0.77(0.69,0.87) | <0.001 | | 0.78(0.69,0.87) | <0.001 | | 0.78(0.66,0.93) | 0.01 |
| Categories |  |  | |  |  | |  |  |
| Q1 | 1.00(Reference) |  | | 1.00(Reference) |  | | 1.00(Reference) |  |
| Q2 | 0.81(0.66,1.00 | 0.05 | | 0.81(0.65,1.00) | 0.05 | | 0.83(0.64,1.08) | 0.16 |
| Q3 | 0.61(0.47,0.80) | <0.001 | | 0.61(0.47,0.80) | <0.001 | | 0.64(0.46,0.91) | 0.01 |
| Q4 | 0.66(0.50,0.86) | 0.003 | | 0.67(0.51,0.88) | 0.004 | | 0.68(0.46,1.01) | 0.06 |
| P for trend |  | <0.001 | |  | <0.001 | |  | 0.03 |
| Male |  |  | |  |  | |  |  |
| Continuous | 0.94(0.83,1.06) | 0.30 | | 0.96(0.84,1.09) | 0.49 | | 0.97(0.82,1.14) | 0.67 |
| Categories |  |  | |  |  | |  |  |
| Q1 | 1.00(Reference) |  | | 1.00(Reference) |  | | 1.00(Reference) |  |
| Q2 | 0.96(0.72,1.30) | 0.81 | | 0.96(0.71,1.29) | 0.77 | | 0.93(0.68,1.27) | 0.63 |
| Q3 | 0.88(0.67,1.15) | 0.35 | | 0.90(0.69,1.18) | 0.46 | | 0.85(0.63,1.16) | 0.31 |
| Q4 | 0.86(0.64,1.15) | 0.29 | | 0.89(0.67,1.20) | 0.45 | | 0.89(0.64,1.24) | 0.50 |
| P for trend |  | 0.2 | |  | 0.38 | |  | 0.46 |
| P for interaction |  | 0.01 | |  | 0.01 | |  | 0.01 |

Abbreviations: PIR, poverty income ratio; BMI, body mass index; OR, odds ratio; CI, confidence interval.

Model 1 was adjusted for nothing.

Model 2 was additionally adjusted for age.

Model 3 was additionally adjusted for model 2+ race, marital status, education attainment, BMI, PIR, drinking status, smoking status, stroke, hypertension,energy consumption, protein consumption, carbohydrate consumption and fat consumption.


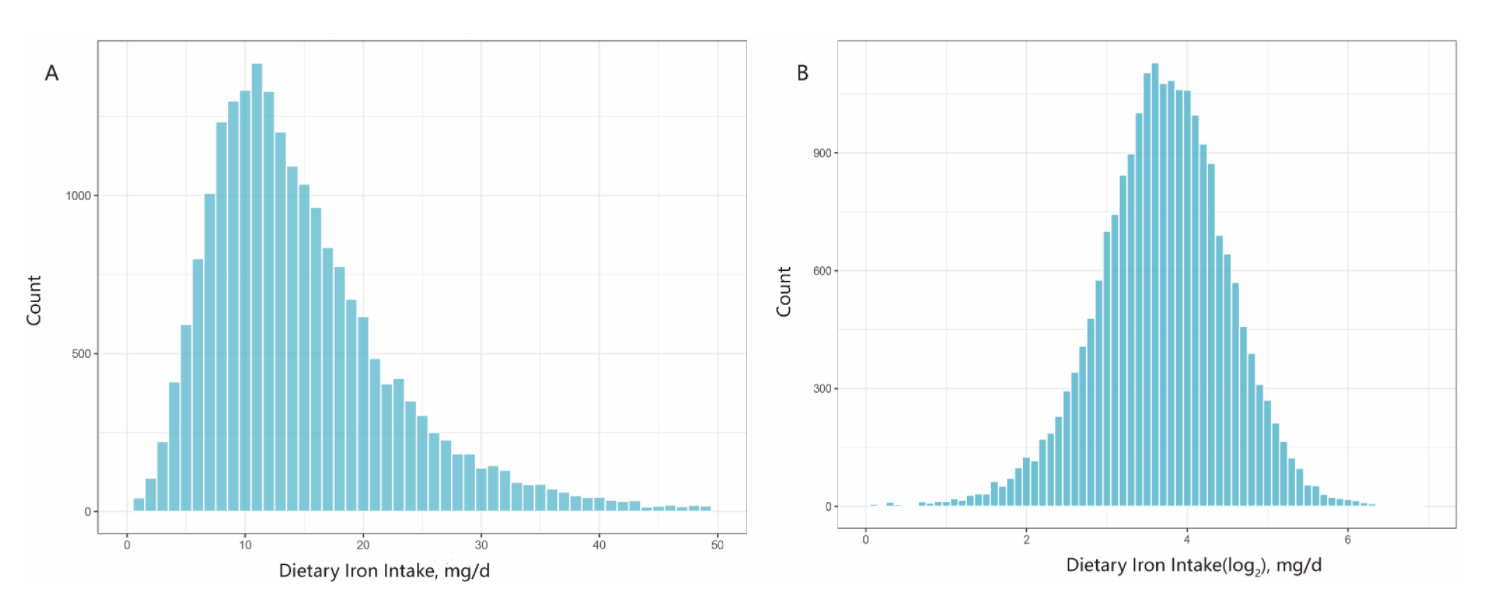


**Supplementary Figure 1.** Distribution of dietary iron intake in the study population. (A) Histogram of untransformed dietary iron intake (mg/d), showing a right-skewed distribution. (B) Histogram of log₂-transformed dietary iron intake, showing a distribution that approximates normality.


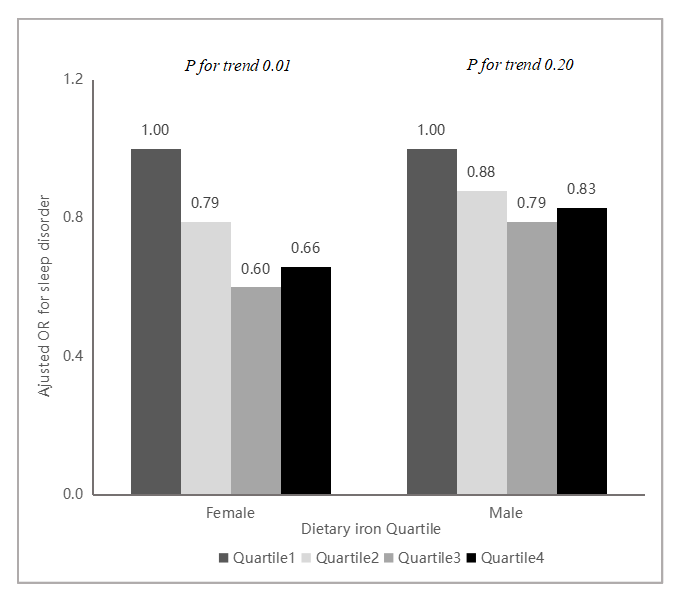


**Supplementary Figure 2.** Relative odds of sleep disorder according to dietary iron quartile among male and female. The adjustment factors included age, race, marital status, education attainment, BMI, PIR, drinking status, smoking status, stroke, hypertension,energy consumption, protein consumption, carbohydrate consumption and fat consumption.


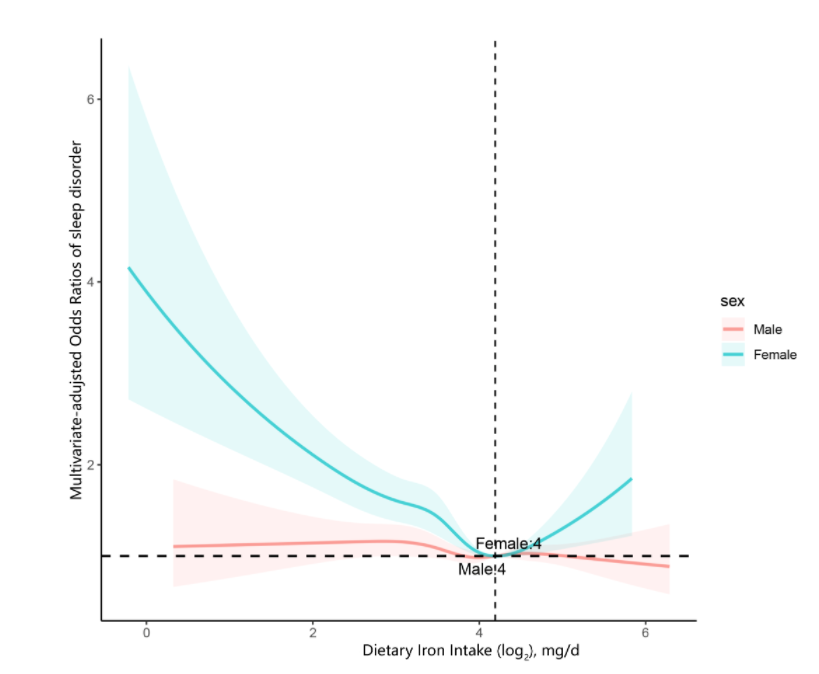


**Supplementary Figure 3.** Association between dietary iron intake and sleep disorder odds ratio among female and male. The model was adjusted for age, race, marital status, education attainment, PIR, BMI, smoking status, drinking status, stroke, hypertension, energy consumption, protein consumption, carbohydrate consumption, and fat consumption.
